# Did the Socio-Economic Gradient in Depression in Later-Life Deteriorate or Weaken during the COVID-19 Pandemic? New Evidence from England Using Path Analysis

**DOI:** 10.3390/ijerph19116700

**Published:** 2022-05-30

**Authors:** Min Qin, Maria Evandrou, Jane Falkingham, Athina Vlachantoni

**Affiliations:** 1 ESRC Centre for Population Change, Faculty of Social Sciences, University of Southampton, Southampton SO17 1BJ, UK; maria.evandrou@soton.ac.uk (M.E.); j.c.falkingham@soton.ac.uk (J.F.); a.vlachantoni@soton.ac.uk (A.V.); 2 Centre for Research on Ageing, Faculty of Social Sciences, University of Southampton, Southampton SO17 1BJ, UK

**Keywords:** older adults, socioeconomic position, depression, COVID-19 pandemic, England

## Abstract

It is well established that there is a socioeconomic gradient in adult mental health. However, little is known about whether and how this gradient has been exacerbated or mitigated by the COVID-19 pandemic. This study aims to identify the modifiable pathways involved in the association between socioeconomic position (SEP) and mental health during the COVID-19 pandemic. The analysis included 5107 adults aged 50+ living in England and participating in the English Longitudinal Study of Ageing Wave nine (2018–2019) and the COVID-19 study (June 2020). Mental health was measured using a shortened version of the Centre for Epidemiologic Studies Depression scale. Path analysis with multiple mediator models was used to estimate the direct effect of SEP (measured by educational qualification and household wealth) on mental health (measured by depression), along with the indirect effects of SEP via three mediators: COVID-19 infection symptoms, service accessibility and social contact. The results show that the prevalence of depression for the same cohort increased from 12.6% pre-pandemic to 19.7% during the first wave of the pandemic. The risk of depression increased amongst older people who experienced COVID-19 infection, difficulties accessing services and less frequent social contact. The total effects of education and wealth on depression were negatively significant. Through mediators, wealth and education were indirectly associated with depression. Wealth also directly affected the outcome. The findings suggest that the socioeconomic gradient in depression among older people may have deteriorated during the initial phase of the pandemic and that this could in part be explained by increased financial hardship, difficulties in accessing services and reduced social contact.

## 1. Introduction

Mental health and many common mental disorders, such as depression and anxiety, are significantly shaped by the socioeconomic environments (neighborhood deprivation, local health and social services inequality) within which people live [1]. Social inequalities are known to be associated with an increased risk of many common mental disorders [1]. Evidence shows that mental health varies according to a socioeconomic gradient across society and that relatively poor and disadvantaged individuals are disproportionately affected [2,3]. 

A review of population surveys in European countries found that higher frequencies of depression and anxiety are associated with low educational attainment, material disadvantage and social isolation [4]. The social causation hypothesis is one fundamental theory to explain the mechanisms underlying the social gradient in mental health. 

This hypothesis postulates that individuals with a lower socioeconomic position are more likely to experience conditions, such as stress and adverse life events, which are in turn thought to precipitate or sustain mental ill-health [3,5]. Previous research shows that the hypothesized processes of social causation can help to explain the inverse relationship between socioeconomic position and depression [6].

Socioeconomic position (SEP) indicators, such as education, occupation and income, are widely used to research physical and mental health inequality [7]. Educational attainment is associated with a healthier lifestyle and more appropriate health care use, which may vary by individuals’ cognitive skills and access to information. Exposure to insecure and poor-quality employment is associated with an increased risk of worsening health. Individuals with low incomes are more likely to refrain from purchasing goods and services that maintain or improve health and more likely to be prevented from participating in social life [7]. 

In addition, in some studies, the association between low income and mental disorders is accounted for by debt [8]. Measuring socioeconomic position in older age groups presents challenges. With many older individuals having retired and not currently economically active, occupation and income might not be considered the best choices of proxies for socioeconomic position amongst this group [9]. Previous research has suggested that wealth, a measure of lifetime accumulation and control over financial resources and assets, can strongly indicate individuals’ current socioeconomic position amongst middle-aged and older people [10]. In the light of these previous studies, the research here employs both educational qualification and wealth as indicators of individuals’ later life socioeconomic position.

The COVID-19 pandemic has impacted individuals in multiple interrelated ways, affecting all aspects of life and posing risks for adverse psychological outcomes [11,12,13,14,15]. Older adults experienced greater adverse effects from the pandemic, including a higher COVID-19 infection rate, severe COVID-19 symptoms and mortality [16,17], concerns about disruptions to their daily routines and access to care [18,19] and difficulty adapting to technologies, such as telemedicine [20,21,22]. 

Other negative effects of the pandemic related lockdown also included altered sleep patterns and physical activity levels [14,23,24]. Although older adults tend to have lower stress reactivity and, in general, better emotional regulation and well-being than younger adults, given the scale and magnitude of the pandemic, attention has been drawn to a potential mental health crisis among older adults [22]. The literature shows that the adverse impacts of the COVID-19 pandemic have not been randomly distributed. Those from the most vulnerable socioeconomic backgrounds and health status groups are likely to be amongst those hit the hardest [12,25,26,27].

The persistence of a socioeconomic gradient in mental health is well established, with empirical evidence pointing towards a negative association between SEP (education and income) and the likelihood of depression [28]. Little, however, is known about whether and how this gradient has been exacerbated or mitigated during the COVID-19 pandemic among older adults. Recent studies show a consensus that the COVID-19 pandemic has worsened depression among adults [29,30,31]. However, the evidence on the relationship between SEP and depression across different studies was inconclusive. Some studies found evidence of a negative association [29], whilst others suggested that there was no association [31] or even a positive relationship [32]. 

Recent research in the US found that lower economic resources (income and savings) were associated with a higher risk of depression symptoms during COVID-19 but that education was not significantly associated with the depression symptoms once economic resources and exposure to stressors were controlled for [29]. A population-based longitudinal study in Norway reported no association between education or income with depression but substantial and equally high increases in depressive symptoms across socioeconomic status [31]. 

A further study in the US found a positive association, with individuals with higher education experiencing a greater increase in depressive symptoms during the COVID-19 pandemic than those with lower education [32]. However, the reasons why individuals with higher educational attainment experienced a greater decline in mental well-being was not clear. Other risk factors associated with depression in these studies included being female, COVID-19 induced stressors (e.g., the death of someone close to you owing to COVID-19 and financial hardship) and experiencing unmet healthcare needs [29,30].

This study aims to contribute to the literature by investigating the potential pathways between individuals’ socioeconomic position and depression. The objective here is to estimate the relative contribution of individuals’ education and wealth through three potential mediators—COVID-19 infection, access to services and social contact—on their report of depression during the pandemic. We formulate the following research questions:

RQ1. How is later life socioeconomic position associated with mental health during the pandemic?

RQ2. What are the mediating pathways through which socioeconomic position influences mental health?

First, we hypothesize that older people with a lower socioeconomic position in terms of their education and household wealth are more likely to experience poor mental health, measured through their report of depression. Second, we hypothesize that the possible mediating pathways through which socioeconomic position influences mental health include:

(a) a higher chance of coronavirus infection,
(b) greater difficulties in access to social services and
(c) reduced social contact.

These hypotheses reflect the fact that individuals with lower socioeconomic positions have, on average, experienced a higher burden of disability and chronic diseases over their life course; as a result, they might have a higher risk of COVID-19 infection disease [16] than those with a higher socioeconomic position (via mediator a). A German nationwide study [33] also found a higher level of viral exposure in low-socioeconomic settings. Individuals with a lower socioeconomic position might also experience greater barriers in accessing transport [34], or digital devices (internet, computer, smartphone, etc.) [35,36,37] and therefore face more difficulties in accessing needed services during lockdown (via mediator b). Finally, we hypothesize that individuals’ lower socioeconomic position would have affected their ability to maintain frequent contact with families and friends outside their household (via mediator c).

## 2. Materials and Methods

### 2.1. Study Design and Setting

The study design relies on secondary data analysis. This includes individuals aged 50 years or older living in England who participated in the English Longitudinal Study of Ageing (ELSA) Wave nine collected in 2018–2019 [38] and who were followed up in the COVID-19 study in June–July 2020 [39]. The ELSA is a nationally representative sample of men and women aged 50 years and older living in England. The ELSA started in 2002, collecting data every two years using face-to-face computer-assisted personal interviews held in participants’ homes and a self-completion questionnaire. 

The study sample is periodically refreshed with new participants to maintain the complete age profile from 50 years and older. The most recent full wave of data collection was Wave nine (in 2018–2019) [38]. In June 2020, an ELSA sub-study was conducted to assess the participants’ experiences during the COVID-19 pandemic [39]. The UK commenced a strict lockdown with orders to stay at home on 23 March 2020, and many restrictions remained in place, though these rules were somewhat relaxed in May and June 2020. Invitations were issued to registered ELSA participants to participate in online or computer-assisted telephone interviews.

In the ELSA datasets, each interviewed respondent has a unique individual ID. Using this we merged the two datasets (ELSA COVID-19 study and Wave nine), linking the data from the COVID-19 study with selected measures collected in Wave nine, including the respondents’ educational qualifications, household wealth and pre-pandemic score on the eight-item Centre for Epidemiological Depression (CED) Scale. Waves 1–9 of the ELSA were approved through the National Research Ethics Service, and the COVID-19 study was approved by the University College London Research Ethics Committee (0017/003). All participants provided informed consent [38,39].

### 2.2. Participants

**Figure A1:**
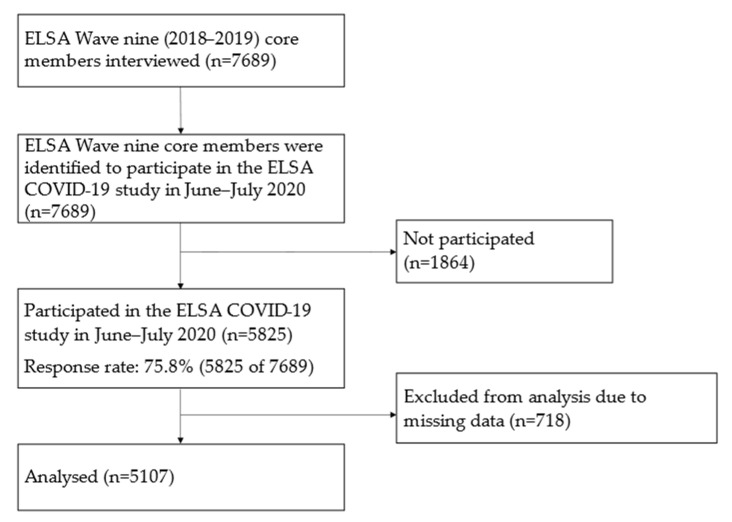
The enrolment of respondents and the selection procedure.

Five thousand eight hundred and twenty-five (5825) core members of ELSA participated in the ELSA COVID-19 study. The inclusion criteria are individuals aged 50 years or older, living in England who participated in the ELSA COVID-19 study in June–July 2020. These respondents also participated in the ELSA Wave nine in 2018–2019. We then excluded respondents with missing data for CED scale in either the ELSA COVID-19 study or in the pre-pandemic Wave nine or who had missing data for their educational qualifications or household wealth. The final analytical sample comprised 5107 participants. The overall mean age was 67.1 years (SD: 10.4; range: 52–95), and 52.8% were female. A flowchart showing the enrollment of respondents and selection procedure is presented in Appendix A Figure A1.

### 2.3. Measures

#### 2.3.1. Measures from the ELSA COVID-19 Study

The outcome variable was depression, measured using a shortened eight-item version of the CED scale in the ELSA COVID-19 study. The original CED scale is a 20-item self-report instrument designed to assess the current levels of depressive symptoms (within the past week) in the general population [40]. An eight-item version of the CED scale was shown to be a valid and reliable instrument for screening depression among older adults in 24 European countries, including the United Kingdom [41]. The scale has a single factor structure and nomological validity [41]. 

In the ELSA COVID-19 study, respondents responded to eight questions regarding their feelings during the past week by answering “yes” or “no” to the following items: (1) you felt depressed, (2) you felt that everything you did was an effort, (3) your sleep was restless, (4) you were happy, (5) you felt lonely, (6) you enjoyed life, (7) you felt sad, and (8) you could not get going. The Cronbach’s alpha of this eight-item CED scale is 0.82, indicating good internal consistency. A depressive symptom score was assigned by totaling all item scores after reversing the questions of the positive mood (Questions 4 and 6). The possible range of scores was 0–8. A cut-off of four or more of the scores was used to identify the caseness of depression. This threshold has been used to identify the prevalence of clinically significant symptoms of depression in previous studies [27].

The path analysis included three mediators. The first mediator is coronavirus infection symptoms. It was coded as 1 for respondents who reported having had two or more core symptoms of COVID-19 as defined by National Health Service (NHS) England (i.e., high temperature, new continuous cough and loss or change in the sense of smell or taste) and 0 for those who had less than two core symptoms. Due to the absence of antigen tests for infection in the population, experiencing two or more core COVID-19 symptoms was used as a proxy for a direct experience of COVID-19 infection. 

The second mediator is service accessibility. In the survey, respondents were asked how easy or difficult it was to get to a bank or cash point, supermarket, hospital or pharmacy, since the coronavirus outbreak. Accessibility difficulty was defined as reporting difficulties getting to, or unable to go to, any of the listed places. This variable was coded as 1 for respondents with any accessibility difficulty and 0 for those without difficulty. The third mediator is social contact. 

The survey asked participants about contact with family outside the household and friends by telephone, video call, email and letter in the past month. Following the approach used in a previous study [27], respondents were defined as having frequent contact if they reported contact with family or friends at least once a week by phone/video-calling or email/letters. A social contact score was then constructed counting the number of these frequent contacts, ranging from 0 to 4. This measurement scale was continuous, with a higher score indicating more social contact.

Demographic variables (age and gender) were considered as potential confounders [15,26] when examining the relationship between individuals’ socioeconomic position and depression. Age was measured as a continuous variable. Gender was a categorical variable—coded 0 for males and 1 for females.

#### 2.3.2. Measures from the ELSA Wave Nine

Socioeconomic variables included the individuals’ educational qualifications and household wealth; both measured on an ordinal scale. Educational qualifications had three categories, coded 0 for less than O-level or equivalent, 1 for O-level or equivalent and 2 for A-level or higher. ‘O-level’ was the qualification taken by this cohort at age 16 (now replaced by GCSE) and maybe thought of as equivalent to a completion of secondary school certificate. ‘A’ levels are the qualification taken at age 18 and are required for University entrance. 

Household wealth was assessed using quintiles of the total net non-pension household wealth, a summary measure of the value of financial, physical and housing wealth owned by the household (i.e., a single respondent or a responding couple along with any dependent individual) minus any debt. The household wealth quintile was coded 0 for the lowest quintile, 1 for the second, 2 for the third, 3 for the fourth and 4 for the highest. The data depositors generated the estimation of this variable. This measurement has been used in previous studies and was found to be more strongly associated with mortality among older people in England than were other measures of socioeconomic position [10].

Pre-pandemic depression was also a potential confounder [15,26] and assessed using the same eight-item CED scale measured in the ELSA Wave nine. Questions on the eight-item CED Scale in the ELSA Wave nine were consistent and comparable with those in the ELSA COVID-19 study. Again, a depressive symptom score was assigned by totaling all item scores after reversing the questions of the positive mood and a cut-off of four or more of the scores was used to identify the caseness of depression. This measurement coded 0 for none-caseness and 1 for pre-pandemic depression caseness.

Variables, such as the respondents’ educational qualifications, household wealth and pre-pandemic CED, were derived from the ELSA Wave nine data, and all other variables were derived from the ELSA COVID-19 study data. This ensured that the socioeconomic variables and pre-pandemic depression were measured before the COVID-19 pandemic when the outcome variable was measured. As noted in the study design and setting section, this study used secondary survey data from the ELSA Wave nine and the COVID-19 study. 

All demographic, socioeconomic and CED scale variables were collected using standardized survey instruments. Most of the data coding and editing was conducted by highly-trained interviewers in the field. Some additional checks related to inconsistencies in the data were conducted after the interview by the survey team. Although data screening was conducted in the field and after the interview [38,39], it is unclear whether data were screened for click-through behaviors or keystroke analysis. The survey data and documents, including questionnaires, are available online from UK Data Service [38,39].

### 2.4. Statistical Analysis

Univariate analysis was used to describe the sample characteristics. Continuous data samples are described using the n, mean and standard deviation (SD), whilst categorical characteristics are described using the n and percentage. Bivariate analysis was then used to assess the strength of the association of the characteristics with depression, using a *t*-test for continuous data and Pearson chi-square tests for categorical variables. Finally, multivariate analysis was conducted using the path analysis method. Path analysis and mediation are commonly used in the social sciences. Mediation occurs when an independent variable X affects a dependent variable Y partly or completely through an intermediate variable Z [42]. 

Path analysis goes beyond regression in that it allows for the analysis of more complicated models. Multiple mediator models were applied with age, gender and pre-pandemic mental health adjusted probit regression to estimate the direct effect of individuals’ socioeconomic position (measured by their highest educational qualification and household wealth) on their mental health (measured by their report of depression) and the indirect effects of SEP via three mediators: COVID-19 infection symptoms, service accessibility and social contact. The advantage of path (mediation) analyses is that it allows researchers to assess the relative magnitude of different pathways and mechanisms by which exposure may affect an outcome [42,43].

A series of sensitivity analyses were conducted to investigate (i) whether differences between socioeconomic positions were due to the socioeconomic measures used and (ii) whether these differentials reflected the fact that the study sample included those younger than 65, many of whom were still in paid employment. The analyses were repeated (i) using income instead of household wealth and (ii) the models were run separately for respondents aged 65 and above and those aged 50–64. All analyses were performed using Mplus 8 [44].

## 3. Results

### 3.1. Sample Characteristics and Bivariate Analysis Results

Information on the sample characteristics is shown in Table 1. Over one-third had an A-level or higher educational qualification, and less than one-third had less than an O-level qualification. The level of depression caseness for the same cohort increased seven percentage points from 12.6% pre-pandemic to 19.7% during the COVID-19 pandemic.

Bivariate analysis (Table 1) shows the association between the independent variables with the dependent variable, depression. One in five respondents reported depression (19.7%). There was a clear socioeconomic gradient in the prevalence of depression, with older persons in the highest quintile of wealth reporting the lowest proportion of depression (13.6%), while those in the lowest quintile had the highest proportion (31.4%). Similarly, 15.8% of respondents with an A-Level or higher educational qualification reported depression, compared to 23.8% of those with less than an O-Level or equivalent educational qualification. The prevalence of depression was higher among respondents who reported two or more core COVID-19 symptoms, difficulty accessing services, or less social contact. Moreover, age, gender and pre-pandemic depression were all associated with depression.

### 3.2. Path Analysis Results

The results (Table 2) show that less-educated older people and those from poorer households had a higher likelihood of being depressed. Education showed only indirect effects but no direct effect. Wealth, however, did have both direct and indirect effects, with the direct effects accounting for 84.7% (−0.083/−0.098) of the total effects on depression.

**Figure 1:**
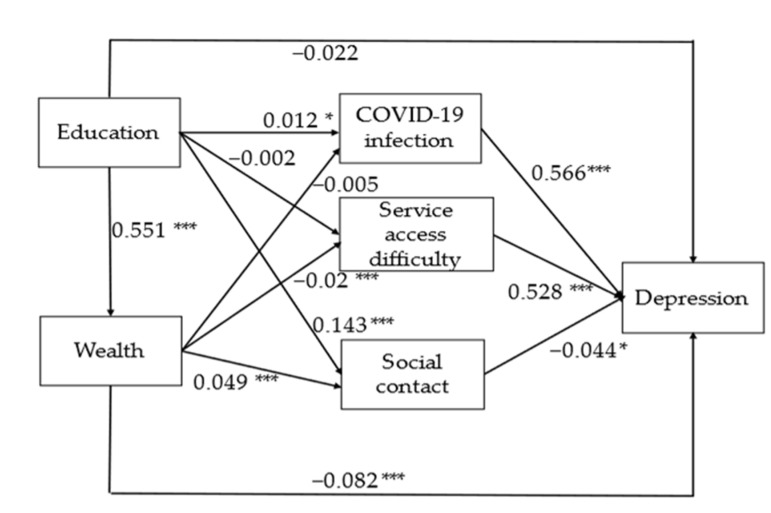
Standardized probit coefficients for the direct effects for the final full structural model, on depression (*n* = 5107, aged 50+ years). Model adjusted by age, gender and pre-pandemic depression. *** *p* < 0.001 and * *p* < 0.05.

Three mediators were identified: COVID-19 infection, service access difficulty and social contact (Figure 1). Older people with COVID-19 symptoms and those reporting difficulties accessing services, including banks, supermarkets and hospitals, had a higher chance of reporting depression. In contrast, those with more frequent contact with families or friends outside the household had a lower chance of reporting.

Through these mediators, two mediation pathways between wealth and depression can be identified (Table 2 and Figure 1). The first pathway is that older people from wealthier households were less likely to be depressed because they were less likely to experience difficulties accessing services. The second pathway is that older people from wealthier households were less likely to be depressed because they had more frequent social. Looking at the overall indirect effects on wealth, the pathway capturing service access difficulty accounted for the majority of these (66.7% (−0.010/−0.015)), whilst social contact accounted for a smaller share (13.3% (−0.002/−0.015)).

The association between education and depression shows more complex pathways (Table 2 and Figure 1). First of all, older people with higher levels of educational attainment were likely to also have higher household wealth and, in turn, a lower likelihood of depression. Education also indirectly affected depression via wealth and service access difficulty. Secondly, those older people with higher educational attainment were more likely to report frequent social contact and reduced depression. Interestingly, the path analysis also highlights that individuals with higher levels of educational attainment had a higher chance of reporting COVID-19 symptoms and a higher chance of depression. Table 2 shows that all indirect effects of education on depression accounted for 71.1% (−0.054/−0.076) of the total effects. 

Once these factors were taken into account, the direct effect of education on depression was no longer significant. Looking at the indirect effects, the pathways via wealth accounted for 83.3% (−0.045/−0.054). Other pathways accounted for a smaller share. The goodness-of-fit indicators confirm that the models were a good fit to the data. The likelihood-ratio Chi-square is highly significant (*p* < 0.001). The root-mean square error of approximation (RMSEA) equals 0.04, the comparative fit index (CFI) equals 0.985, and the Tucker–Lewis index (TLI) equals 0.85.

The sensitivity analyses demonstrated that the results are robust. The results using income instead of household wealth were similar to the main path analyses (Appendix A Table A1). The results also showed no meaningful differences when running the model separately among participants older than 65 (Appendix A Table A2) and those aged 50–64 (Appendix A Table A3).

## 4. Discussion

This study aims to shed light on whether and how the socioeconomic gradient in mental health has been exacerbated or mitigated during the pandemic, investigating the impact of infection itself and a range of factors associated with policy measures introduced to combat the pandemic, including lockdown. Using newly collected national representative survey data, path analysis was conducted quantifying the mediation effect of COVID-19, distinguishing between direct (COVID-19 symptoms) and indirect (social isolation) impacts of the pandemic on the association between the socioeconomic position and older people’s mental health. 

The study highlights that depression in the same cohort of older people increased during the first wave of the pandemic in England. The total effects of education and wealth on depression were negatively significant. The association between SEP and depression were partially mediated by COVID-19 infection, accessing services and social contact. The findings suggest that the socioeconomic gradient in de-pression among older people may have deteriorated during the initial phase of the pandemic and that this could in part be explained by increased financial hardship, difficulties in accessing services and reduced social contact.

The results support the first hypothesis and confirm that there are inequalities in mental health in later life and that these were exacerbated during the COVID-19 pandemic. In line with other studies [29], older people from poorer households were more likely to report depressive symptoms. Not surprisingly, wealth had a direct negative association with depression. Older people from less wealthy households may have been more likely to experience financial hardship during the pandemic and/or worry about their future ability to meet their needs. 

Less well-off older people may also be more likely to experience a disproportionate impact from rising debt, long-term unemployment and a lack of financial security, all of which are known to be associated with poorer mental health [11,45]. Education showed no direct effect on depression; however, the results provide evidence of its indirect effect via other mediators. Early life education is vital in building emotional resilience and affects a range of life course outcomes which reduce the risk of poor mental health, such as employment, income, accumulated wealth and social integration [7]. This finding is consistent with other studies reporting no direct association between education and depression symptoms, considering economic resources and exposure to stressors in the statistical model [29].

The results partly support the second hypothesis. The association between SEP and depression is mediated through difficulties in accessing social services and reduced social contact. As hypothesized, several mediation pathways between socioeconomic position and depression could be clearly identified (Table 2 and Figure 1). Older people from less wealthy households were more likely to be socially isolated and to report experiencing difficulties accessing services, such as banks, supermarkets and hospitals, during the first few months of pandemic, up to June 2020. 

In turn, older persons from less wealthy households were more likely to report depression. In addition, less wealthy older people had less frequent contact with families and friends outside the household through phone or video-calling or written contact, which again was associated with a higher chance of depression. Similarly, highly educated older people had more frequent social contact and reduced depression. Individuals’ education also indirectly affected their report of depression via wealth and service access difficulty. 

These pathways are in line with previous studies suggesting that adults with a lower SEP are more likely to experience COVID-19 induced stressors [46]. Such stressors increase individuals’ chances of depression [29]. These results provide a deeper understanding of the complex relationship between socioeconomic position and depression and support identifying more effective interventions.

The study confirms that the socioeconomic gradient in the prevalence of depression in later life widened during the COVID-19 pandemic. One possible explanation lies in the increased experience of social isolation resulting from physical distancing to reduce coronavirus transmission. A survey showed that over half of older adults reported difficulties accessing essential supplies during the first lockdown due to fears about their physical vulnerability to the virus [37]. An increasing number of service providers changed from in-person to virtual provision of information and services. 

Studies show that older people, especially those with lower socioeconomic positions, are more likely to be excluded from digital modes of communication, including the internet and smartphones. Those older people without access to the internet are likely to become increasingly disadvantaged as the internet’s societal pervasiveness progresses [36]. Moreover, even those older people who are ‘connected’ may face difficulties accessing online services, banking, or shopping and be more reluctant to take up offers of remote consultations with GPs and other health care providers. 

A qualitative study found that digital skill levels, affordability of technology, disabilities and language barriers are all associated with older people’s digital exclusion [47]. Education and wealth are among many factors contributing to digital exclusion. Older adults with higher educational and economic status were more likely to use the internet and adopt newer technologies [20,36].

An unexpected finding from the study was that the path analysis highlighted that older people with higher education had a higher chance of reporting COVID-19 symptoms and a higher chance of depression. This relationship warrants further exploration. One of the possible explanations might be that those older people with a higher level of education may be more likely to still be working, either as employees or self-employed, which in turn results in an increase in the risk of exposure to coronaviruses infection. 

Factors related to mental health are multidimensional [1], it might be possible that some other factors, such as altered sleep patterns and physical activity levels that are likely to change during the pandemic [23,24], may link with education as well as depression. A positive association between education and depression was reported in a previous study [32], although the reasons behind this finding were not clear.

This study makes a unique contribution to our understanding of the socioeconomic gradient in mental health, particularly during the COVID-19 pandemic. The strengths of this study include its large sample size and the ability to explore multiple competing hypotheses using the ELSA data, a unique resource for studying the relationship between socioeconomic position and mental health. By investigating the mechanisms through which older individuals’ socioeconomic position influences the likelihood of experiencing depression, the study has provided new insights into how older people from lower socioeconomic groups are at a higher risk of depression during the pandemic. Such valuable information can inform the design of interventions to protect mental health during the pandemic.

## 5. Limitations

However, the study is not without limitations. Most importantly, it focused on only three types of mediators, reflecting those relating to health, social connections and basic needs; however, the models do not rule out the possibility of omitted mediators and confounders which might bias the results. For instance, impaired sleep quality could be a potential confounder between socioeconomic position and depression. As wealth is correlated with impaired sleep quality [14], this risk factor is also associated with depression [48]. 

Without considering impaired sleep quality, the effects of confounding may result in an overestimate of the association between wealth and depression. Future studies are needed to investigate the specific stressors involved in the association between individuals’ SEP and depression. Furthermore, the study only followed individuals during the initial stages of the pandemic, with the data being collected in June 2020. 

Although the strictures of the first lockdown in the UK were beginning to be lifted in June 2020, the COVID-19 pandemic has continued to affect people’s daily lives. Further national lockdowns were introduced in September 2020 and again in early 2021 as infections rose, with all restrictions only being lifted in February 2022 under the ‘Living with COVID’ plan [49]. Further research is required to assess how the experience of long-term adversity during the COVID-19 pandemic relates to long-term mental health consequences.

## 6. Conclusions

The COVID-19 pandemic is a global public health crisis. To our knowledge, this study is the first to investigate multiple pathways in the link between socio-economic position and depression in later life during the pandemic, with the results suggesting that the socio-economic gradient in the prevalence of depression among older people has deteriorated. Financial hardship, difficulties in accessing services and reduced social contact could all contribute to this. As the pandemic continues to spread worldwide, the COVID-19 virus and social responses to the pandemic pose a continuing risk to good mental health amongst older persons. 

Policy makers and practitioners need to take action to reduce social isolation and digital exclusion, whilst improving financial protections for the poorest older people. Otherwise, there is a danger that socioeconomic inequalities in mental health in later life will continue to widen. Older individuals with a disadvantaged socioeconomic position are more likely to require hospital or social care and have consequently been more affected by cancellations. 

In order to address the impact of missed care appointments, policies should prioritize vulnerable individuals and ensure that they avoid disruptions during the pandemic, which can exacerbate long-standing health inequalities [19]. The best approach to gaining digital skills for the majority of older people is ongoing support according to their needs and capacity. Those who cannot or do not wish to use the internet should still be able to access services and support that suits them [50].

## Figures and Tables

**Table 1:** Characteristics of the respondents and the bivariate association between characteristics and depression caseness (*n* = 5107, aged 50+ years).

	Number (% among All Respondents)	Row % of Depression Caseness	*p* Value
All respondents	5107 (100.0)	19.7	
Age	Mean age of all respondents 67.1	Mean age of depressed 66.4; Mean age of not depressed 67.2	0.026
Gender			<0.001
Male	2211 (47.2)	14.5	
Female	2896 (52.8)	24.4	
Education			<0.001
Less than O-level or equivalent	1283 (30.0)	23.8	
O-level or equivalent	1721 (35.2)	20.2	
A-level or higher	2103 (34.8)	15.8	
Household wealth quintile			<0.001
Lowest	683 (20.1)	31.4	
2	869 (19.2)	22.9	
3	1145 (21.0)	17.3	
4	1208 (20.3)	13.6	
Highest	1202 (19.4)	13.6	
Two or more core COVID-19 symptoms			<0.001
No	4514 (87.0)	16.8	
Yes	593 (13.0)	39.1	
Difficulty in accessing services			<0.001
No	3658 (71.8)	14.1	
Yes	1449 (28.2)	34.2	
Social contact score			0.002
0	172 (3.9)	27.7	
1	314 (6.2)	25.2	
2	942 (19.4)	20.6	
3	688 (13.5)	18.8	
4	2991 (57.0)	18.5	
Pre-pandemic depression			<0.001
No	4574 (87.4)	13.8	
Yes	533 (12.6)	60.6	

Weighted %, non-weighted number of respondents. *p*-value based on *t*-test for compare mean age of depressed and mean age of not depressed and Pearson chi-square tests for the association between all other characteristic variables and depression caseness.

**Table 2:** Standardised probit coefficients for the direct and indirect effects for the final full structural model (*n* = 5107, aged 50+ years).

	Coefficients (Standard Error)
Education direct effect	−0.022 (0.027)
Education > COVID-19 infection	0.007 (0.004) *
Education > service access difficulty	−0.001 (0.004)
Education > social contact	−0.006 (0.003) *
Education > wealth	−0.045 (0.009) ***
Education > wealth > COVID-19 infection	−0.002 (0.001)
Education > wealth > service access difficulty	−0.006 (0.002) **
Education > wealth > social contact	−0.001 (0.001)
Total indirect effects of education	−0.054 (0.011) ***
Total effects of education	−0.076 (0.027) **
Wealth direct effects	−0.083 (0.017) ***
Wealth > COVID-19 infection	−0.003 (0.002)
Wealth > service access difficulty	−0.010 (0.003) **
Wealth > social contact	−0.002 (0.001) *
Total indirect effects of wealth	−0.015 (0.004) ***
Total effects of wealth	−0.098 (0.017) ***

Model adjusted by age, gender and pre-pandemic depression. *** *p* < 0.001, ** *p* < 0.01, and * *p* < 0.05. Model fit: The likelihood-ratio Chi-Square Test of Model Fit *p* < 0.001, RMSEA = 0.040, CFI = 0.985, and TLI = 0.850.

## Data Availability

The survey data used for this study is available from UK Data Service https://ukdataservice.ac.uk/ upon application (accessed on 1 September 2021).

## Appendix A

**Table A1:** Standardized probit coefficients for the direct and indirect effects for the final full structural model (*n* = 5107, aged 50+ years, income quintile used).

	Coefficients (Standard Error)
Education direct effect	−0.036 (0.027)
Education > COVID-19 infection	0.004 (0.003)
Education > service access difficulty	−0.003 (0.004)
Education > social contact	−0.007 (0.003) *
Education > income	−0.03 (0.009) ***
Education > income > COVID-19 infection	0.001 (0.001)
Education > income > service access difficulty	−0.004 (0.002) **
Education > income > social contact	−0.001(<0.001)
Total indirect effects of education	−0.04 (0.011) ***
Total effects of education	−0.076 (0.027) **
Income direct effect	−0.058 (0.016) ***
Income > COVID-19 infection	0.002 (0.002)
Income > service access difficulty	−0.009 (0.003) **
Income > social contact	−0.002 (0.001)
Total indirect effects of income	−0.008 (0.004) *
Total effects of income	−0.066 (0.016) ***

Model adjusted by age, gender and pre-pandemic depression. *** *p* < 0.001, ** *p* < 0.01 and * *p* < 0.05. Model fit: The likelihood-ratio Chi-Square Test of Model Fit *p* < 0.001, RMSEA = 0.04, CFI = 0.983 and TLI = 0.83. Note: Income is the sum of employment income, self-employment income, state benefit income, state pension income, private pension income, asset income and other income and adjusted for benefit unit size [38].

**Table A2:** Standardized probit coefficients for the direct and indirect effects for the final full structural model (*n* = 3818, aged 65+ years).

	Coefficients (Standard Error)
Education direct effect	−0.038 (0.031)
Education > COVID-19 infection	0.008 (0.004)
Education > service access difficulty	−0.002 (0.005)
Education > social contact	−0.003 (0.003)
Education > wealth	−0.042 (0.010) ***
Education > wealth > COVID-19 infection	−0.001 (0.001)
Education > wealth > service access difficulty	−0.004 (0.002) *
Education > wealth > social contact	−0.001 (0.001)
Total indirect effects of education	−0.046 (0.011) ***
Total effects of education	−0.084 (0.030) ***
Wealth direct effect	−0.082 (0.019) ***
Wealth > COVID-19 infection	−0.002 (0.002)
Wealth > service access difficulty	−0.007 (0.004) *
Wealth > social contact	−0.001 (0.002)
Total indirect effects of wealth	−0.011 (0.005) *
Total effects of wealth	−0.092 (0.019) ***

Model adjusted by age, gender and pre-pandemic depression. *** *p* < 0.001 and * *p* < 0.05. Model fit: The likelihood-ratio Chi-Square Test of Model Fit *p* < 0.001, RMSEA = 0.028, CFI = 0.991 and TLI = 0.914.

**Table A3:** Standardized probit coefficients for the direct and indirect effects for the final full structural model (*n* = 1289, aged 50–64 years).

	Coefficients (Standard Error)
Education direct effect	0.006 (0.061)
Education > COVID-19 infection	0.005 (0.008)
Education > service access difficulty	0.004 (0.009)
Education > social contact	−0.009 (0.006)
Education > wealth	−0.059 (0.019) **
Education > wealth > COVID-19 infection	−0.002 (0.002)
Education > wealth > service access difficulty	−0.008 (0.004) *
Education > wealth > social contact	0.002 (0.002)
Total indirect effects of education	−0.068 (0.023) **
Total effects of education	−0.062 (0.06)
Wealth direct effect	−0.082 (0.019) ***
Wealth > COVID-19 infection	−0.004 (0.004)
Wealth > service access difficulty	−0.014 (0.006) *
Wealth > social contact	0.003 (0.003)
Total indirect effects of wealth	−0.015 (0.008)
Total effects of wealth	−0.116 (0.032) ***

Model adjusted by age, gender and pre-pandemic depression. *** *p* < 0.001, ** *p* < 0.01 and * *p* < 0.05. Model fit: The likelihood-ratio Chi-Square Test of Model Fit *p* < 0.001, RMSEA = 0.025, CFI = 0.994 and TLI = 0.940.
